# Benzene-Induced Aberrant miRNA Expression Profile in Hematopoietic Progenitor Cells in C57BL/6 Mice

**DOI:** 10.3390/ijms161126001

**Published:** 2015-11-12

**Authors:** Haiyan Wei, Juan Zhang, Kehong Tan, Rongli Sun, Lihong Yin, Yuepu Pu

**Affiliations:** Key Laboratory of Environmental Medicine Engineering of Ministry of Education, School of Public Health, Southeast University, Nanjing 210009, China; 230129497@seu.edu.cn (H.W.); 220122806@seu.edu.cn (K.T.); 230109509@seu.edu.cn (R.S.); lhyin@seu.edu.cn (L.Y.)

**Keywords:** benzene, miRNA, hematopoietic progenitor cells

## Abstract

Benzene is a common environmental pollutant that causes hematological alterations. MicroRNAs (miRNAs) may play a role in benzene-induced hematotoxicity. In this study, C57BL/6 mice showed significant hematotoxicity after exposure to 150 mg/kg benzene for 4 weeks. Benzene exposure decreased not only the number of cells in peripheral blood but also hematopoietic progenitor cells in the bone marrow. Meanwhile, RNA from Lin^−^ cells sorted from the bone marrow was applied to aberrant miRNA expression profile using Illumina sequencing. We found that 5 miRNAs were overexpressed and 45 miRNAs were downregulated in the benzene exposure group. Sequencing results were confirmed through qRT-PCR. Furthermore, we also identified five miRNAs which significantly altered in Lin^−^c-Kit^+^ cells obtained from benzene-exposed mice, including mmu-miR-34a-5p; mmu-miR-342-3p; mmu-miR-100-5p; mmu-miR-181a-5p; and mmu-miR-196b-5p. In summary, we successfully established a classical animal model to induce significant hematotoxicity by benzene injection. Benzene exposure may cause severe hematotoxicity not only to blood cells in peripheral circulation but also to hematopoietic cells in bone marrow. Benzene exposure also alters miRNA expression in hematopoietic progenitor cells. This study suggests that benzene induces alteration in hematopoiesis and hematopoiesis-associated miRNAs.

## 1. Introduction

Benzene, a ubiquitous environmental pollutant, is not only harmful to occupational workers [1,2] but also poses a potential health threat to individuals who smoke cigarettes [3,4] or are living in a newly decorated house [5,6]. As benzene is highly volatile, it is rapidly absorbed and metabolized into various intermediate compounds, including catechol, hydroquinone, and *p*-benzoquinone [7,8]. Researchers found that benzene exposure could cause adverse effects, such as skin irritation, central nervous system depression, immunotoxicity and particularly hematoxicity [9,10]. Diseases of the hematopoietic system associated with benzene exposure include myelodysplastic syndrome, aplastic anemia, leukemia, and lymphoma [11,12,13].

MicroRNAs (miRNAs) may be involved in benzene-induced hematotoxicity, in addition to other well-known mechanisms, such as lipid peroxidation, apoptosis, DNA damage, and stem cell microenvironment disturbance. MiRNAs, which are endogenous non-coding RNA (19–24 nucleotide long), regulate gene expression by way of mRNA degradation or transcriptional inhibition [14] at the transcriptional or post-transcriptional level [15,16]. MiRNAs play important roles in multiple biological processes, including development [17], apoptosis [18,19], cell proliferation and differentiation [20,21], and stem cell division and development [22,23,24]. Alterations in miRNA expression have been found in several diseases, including solid and hematological malignancies [25,26]. However, the number of studies regarding the expression features and functions of miRNAs after exposure to exogenous pollutants is relatively lower than cancer studies [27,28]. Bai *et al.* [29] reported the aberrant miRNA profiles and mRNA expression patterns in peripheral blood cells of chronic benzene-exposed patients. As peripheral blood cells are continuously replenished from a pool of hematopoietic stem and progenitor cells (HSPCs) in bone marrow, we planned to study benzene-induced hematotoxicity and alteration in miRNAs profile in bone marrow HSPCs from benzene-exposed male C57BL/6 mice.

## 2. Results

### 2.1. Body Weight and Organ Coefficient

**Figure 1:**
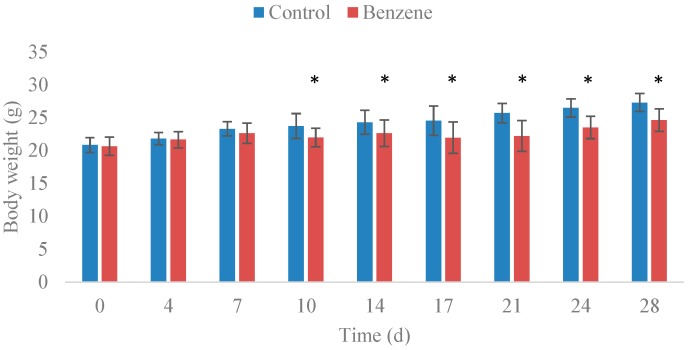
Effect of benzene exposure on body weight (g). The data are presented as the mean ± SD. *n* = 12 for each group. * *p* < 0.05.

The effects of benzene exposure on body weight are shown in Figure 1. After exposure to 150 mg/kg benzene for 10 days, mice showed lower body weight than the control group (*p* < 0.05). The thymus/body weight coefficient significantly decreased, whereas the liver/body weight coefficient significantly increased (Table 1) in the benzene exposure group (*p* < 0.05).

**Table 1:** Effect of benzene exposure on organ coefficient.

Group	Liver/Body Weight	Spleen/Body Weight	Thymus/Body Weight
Control	4.67 ± 0.55	0.38 ± 0.10	0.15 ± 0.03
Benzene	5.46 ± 0.26 *	0.32 ± 0.12	0.12 ± 0.02 *

The data are presented as the mean ± SD. *n* = 12 for each group. * *p* < 0.05.

### 2.2. Hematological Parameters

Table 2 shows the effects of benzene exposure on blood parameters. C57BL/6 mice in the benzene exposure group showed decreased numbers of peripheral white blood cells, red blood cells, and lymphocytes, as well as hemoglobin concentration. By contrast, the MCV significantly increased in the benzene exposure group.

**Table 2:** Blood parameters of mice in benzene exposure and control groups.

Group	WBC (10^9^/L)	RBC (10^12^/L)	Hb (g/L)	Plt (10^9^/L)	Neut (10^9^/L)	Lym (10^9^/L)	MCV (fL)
Control	8.49 ± 1.25	10.11 ± 1.55	149.34 ± 20.53	715.13 ± 137.46	1.35 ± 0.56	6.03 ± 1.35	51.98 ± 1.15
Benzene	3.84 ± 1.17 *	8.97 ± 0.53 *	136.13 ± 8.24	863.72 ± 71.18 *	0.99 ± 0.39 *	2.84 ± 0.85 *	55.9 ± 1.01 *

The data are presented as the mean ± SD. *n* = 12 for each group. * *p* < 0.05.WBC, white blood cells; RBC, red blood cells; Hb, hemoglobin; Plt, platelets; Neut, neutrophils; Lym, lymphocytes; MCV, the mean corpuscular volume.

### 2.3. Flow Cytometric Analysis of HSPCs

After benzene exposure for 4 weeks, we performed flow cytometric to determine enumerations of HSPCs from the bone marrow of C57BL/6 mice. Table 3 shows that the number of Lin^−^c-Kit^+^ cells significantly decreased in benzene-exposed mice. (*p* < 0.05).

**Table 3:** Effect of benzene exposure on the numbers of HSPCs.

Group	Lin^−^ Cells	Lin^−^c-Kit^+^ Cells
Control	16026.64 ± 3774.60	2465.36 ± 546.65
Benzene	12339.45 ± 6566.61	1307.46 ± 584.37 *

The data are presented as the mean ± SD. *n* = 12 for each group. * *p* < 0.05.

### 2.4. Aberrant miRNA Expression Based on Sequencing

**Figure 2:**
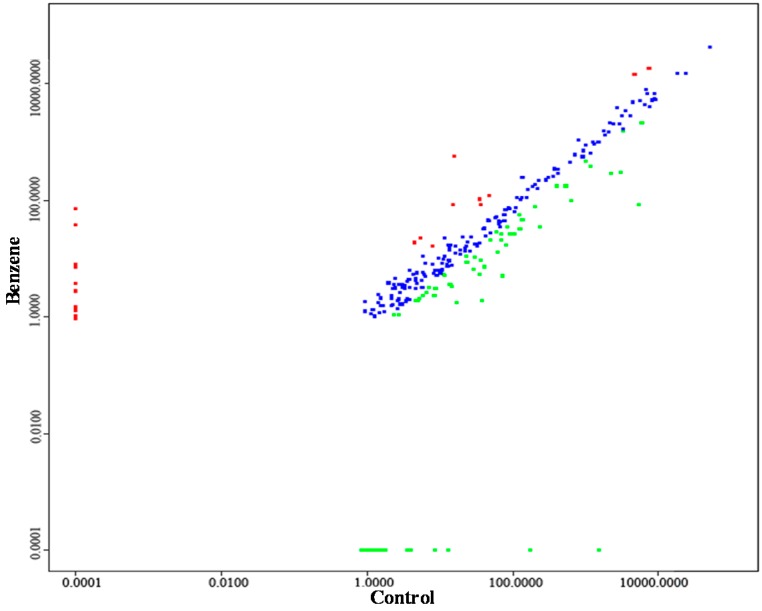
Scatterplot showing the relationship between the benzene exposure group and the control group. Red dots indicate the 2-fold upregulated miRNAs and green dots indicate the 2-fold downregulated miRNAs compared with the control group. Blue dots indicate no differences between two groups. *n* = 3 for each group.

Benzene exposure produced differential expression patterns of miRNAs in C57BL/6 mice (Figure 2). The sequencing data identified 50 differentially expressed miRNAs after benzene exposure (Table 4, *p* < 0.05), of which five miRNAs were overexpressed and 45 miRNAs were downregulated. The upregulated miRNAs included mmu-miR-34a-5p, mmu-miR-129b-5p, mmu-miR-451a, mmu-miR-144-5p and mmu-miR-129b-3p, whereas highly downregulated miRNAs included mmu-miR-100-5p, mmu-miR-99a-5p, mmu-miR-33-5p, mmu-miR-125a-5p, mmu-miR-128-1-5p, mmu-miR-181b-1-3p, mmu-miR-188-5p, mmu-miR-196b-5p, mmu-miR-211-5p, mmu-miR-224-5p, mmu-miR-455-3p, mmu-miR-504-5p, mmu-miR-592-5p, mmu-miR-5107-3p, mmu-miR-5120, and mmu-let-7i-3p.

**Table 4:** Aberrant miRNAs profile of Lin^−^ cells expressed between benzene exposure group and the control group.

miRNA_ID	Control	Benzene	|log_2_ (Fold Change)|	*p*-Value	Significance-Lable	Regulation
mmu-miR-17-3p	83.7974	35.1372	1.253906	0.0114	*	down
mmu-miR-28a-3p	23.3702	8.7843	1.411671	0.0239	*	down
mmu-miR-29a-3p	121.5766	55.9239	1.120329	0.0201	*	down
mmu-miR-29c-3p	1009.316	466.1916	1.114384	0.00865	**	down
mmu-miR-33-5p	1.6325	0	13.9948	0.0475	*	down
mmu-miR-99a-5p	624.7338	97.3232	2.682386	0.000487	**	down
mmu-miR-99b-5p	13.7108	3.5659	1.942975	0.000161	**	down
mmu-miR-100-5p	2226.083	288.6646	2.947041	0.0000316	**	down
mmu-miR-125a-5p	61.9113	12.6981	2.28559	0.000129	**	down
mmu-miR-126a-3p	200.2112	77.4933	1.369379	0.00904	**	down
mmu-miR-126a-5p	30.3297	10.5238	1.527075	0.0106	*	down
mmu-miR-128-1-5p	1.4606	0	13.83427	0.000663	**	down
mmu-miR-141-3p	90.3593	26.266	1.782477	0.000172	**	down
mmu-miR-181a-1-3p	69.7313	20.508	1.76562	0.00506	**	down
mmu-miR-181d-5p	1144.295	380.4057	1.588848	0.0000964	**	down
mmu-miR-181a-5p	5882.768	2099.102	1.486723	0.024	*	down
mmu-miR-181b-1-3p	28.9987	6.5003	2.15741	0.0188	*	down
mmu-miR-181c-3p	134.7541	46.3262	1.540429	0.0112	*	down
mmu-miR-188-5p	0.9368	0	13.19353	0.0147	*	down
mmu-miR-191-5p	3285.317	1517.686	1.114159	0.0473	*	down
mmu-miR-196b-5p	34.9694	5.3101	2.719282	0.000407	**	down
mmu-miR-199b-5p	7.0255	3.2044	1.132547	0.0498	*	down
mmu-miR-203-3p	125.1852	31.8607	1.974214	0.00191	**	down
mmu-miR-211-5p	1.117	0	13.44734	0.00186	**	down
mmu-miR-224-5p	8.4455	0	16.3659	0.000949	**	down
mmu-miR-324-3p	59.3565	28.7012	1.048295	0.0416	*	down
mmu-miR-342-5p	11.1361	5.3923	1.046271	0.0338	*	down
mmu-miR-342-3p	403.5655	175.4169	1.202015	0.00359	**	down
mmu-miR-425-3p	49.5631	20.7827	1.253883	0.024	*	down
mmu-miR-455-5p	103.3616	26.2759	1.975888	0.0167	*	down
mmu-miR-455-3p	14.3486	3.2959	2.122166	0.0034	**	down
mmu-miR-504-5p	3.5127	0	15.10029	0.000458	**	down
mmu-miR-592-5p	71.7493	5.0445	3.830182	0.000744	**	down
mmu-miR-671-3p	8.6863	3.0213	1.523572	0.0323	*	down
mmu-miR-872-5p	68.65	26.0013	1.400676	0.00157	**	down
mmu-miR-874-3p	5.8296	2.3421	1.315594	0.00755	**	down
mmu-miR-1291	5.1552	2.0874	1.304321	0.00577	**	down
mmu-miR-3060-5p	1.1071	0	13.4345	0.000621	**	down
mmu-miR-5104	2.3198	1.0986	1.078334	0.0317	*	down
mmu-miR-5107-3p	1.2706	0	13.63322	0.00982	**	down
mmu-miR-5120	1.1922	0	13.54134	0.0195	*	down
mmu-miR-6988-3p	5.0244	1.9226	1.385893	0.00503	**	down
mmu-miR-7043-3p	22.7206	11.078	1.036303	0.00864	**	down
mmu-miR-7649-5p	8.3457	2.3483	1.829416	0.0346	*	down
mmu-let-7i-3p	1.0463	0	13.35301	0.0185	*	down
mmu-miR-34a-5p	15.3215	83.1308	2.439826	0.0075	**	up
mmu-miR-129b-5p	0	3.7399	15.19071	0.00879	**	up
mmu-miR-451a	4681.085	14232.5	1.604274	0.0241	*	up
mmu-miR-144-5p	46.7118	120.2277	1.36391	0.0251	*	up
mmu-miR-129b-3p	4.4096	18.7862	2.090954	0.0211	*	up

Lin^−^ cells from two mice were pooled as one sample, three samples per group were analyzed for miRNAs profile. *n* = 3 for each group. * *p* < 0.05. ** *p* < 0.01.

### 2.5. Validation of miRNA Sequencing

**Figure 3:**
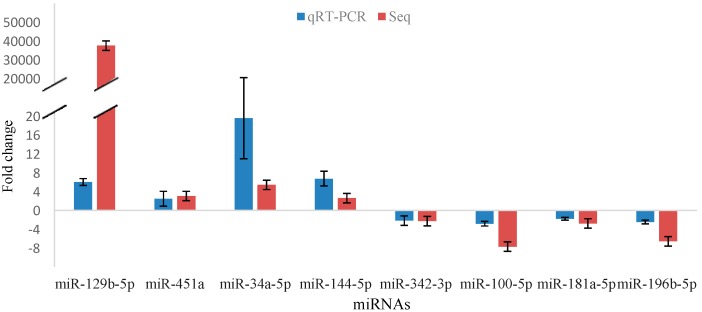
Confirmation of deregulated miRNAs in Lin^−^ cells by qRT-PCR compared with sequencing. The data are presented as the mean ± SD. *n* = 6 for each group.

We performed qRT-PCR to confirm the sequencing results for the expression profile analysis. In agreement with the sequencing data, the expression levels of mmu-miR-129b-5p, mmu-miR-451a, mmu-miR-34a-5p and mmu-miR-144-5p increased in the benzene exposure group, whereas the levels of mmu-miR-342-3p, mmu-miR-100-5p, mmu-miR-181a-5p, and mmu-miR-196b-5p decreased in the benzene exposure group (Figure 3). Although the qRT-PCR results displayed a regulation trend similar to the sequencing data, several minor differences were found in the expression intensities of these two different detection methods. As observed in the sequencing data, mmu-miR-129b-5p showed the highest increase among all detected miRNAs, and the expression of this miRNA increased relatively by six-fold over the control group in qRT-PCR detection. In addition, the expression of mmu-miR-34a-5p increased by five-fold in the sequencing data and by approximately 19.55-fold in qRT-PCR detection.

### 2.6. Aberrant Expression of miRNAs in Lin^−^c-Kit^+^ Cells of Mice Exposed to Benzene

**Figure 4:**
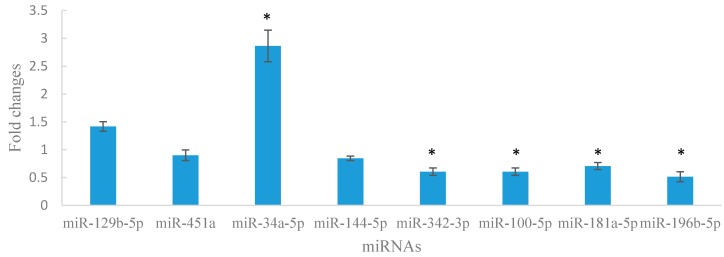
Significantly differential expression of miRNAs in Lin−c^-^Kit^+^ cells of mice exposed to benzene compared with the control group. The data are presented as the mean ± SD. n = 4 for each group. * *p* < 0.05.

We detected the expression of 8 miRNAs (mmu-miR-129b-5p, mmu-miR-451a, mmu-miR-34a-5p, mmu-miR-144-5p, mmu-miR-342-3p, mmu-miR-100-5p, mmu-miR-181a-5p, and mmu-miR-196b-5p) in Lin^−^c-Kit^+^ cells through qRT-PCR. Five of these miRNAs significantly changed in Lin^−^c-Kit^+^ cells of benzene-exposed mice (Figure 4). Similar results were found in Lin^−^ cells of benzene-exposed mice.

## 3. Discussion

As we known, benzene exposure is associated with many serious hematological malignancies [11,12,13]. MiRNAs play an important role in normal biological processes [17,19,20,21,22,23,24] and abnormal hematological malignancies [18,25,26]. However, the implication of miRNAs in benzene-induced hematotoxicity remain unclearly. Furthermore, there is no report on alteration in miRNAs profile in bone marrow HSPCs. Therefore, we plan to study benzene-induced hematotoxicity and alteration in the miRNA profile in bone marrow HSPCs from benzene-exposed mice.

Given the strain and gender differences reported after benzene exposure, we selected male C57BL/6 mice as the animal model for benzene exposure in this study [30,31,32,33,34]. Previous studies confirmed that a model induced with benzene through injection can be easily controlled and does not require the use of expensive equipment [35]. Therefore, we developed a benzene exposure model through subcutaneous injection for 4 weeks.

In this study, body weight evidently decreased after 10 days of benzene exposure. Exposure of mice to benzene led to a significant decrease in thymus/body weight. This significant decrease indicated that immunotoxicity could be induced by benzene exposure in C57BL/6 mice. A large decrease in the number of white blood cells, red blood cells, and lymphocytes, were found after benzene exposure for 4 weeks (Table 2). These results are in accordance with previous studies conducted on mice and benzene-exposed workers [35,36]. Furthermore, the number of various hematopoietic progenitor cells in mouse bone marrow also decreased in the benzene exposure group. The numbers of Lin^−^c-Kit^+^ cells showed significant decrease after benzene exposure. These results are similar to previous data and confirmed that benzene-induced hematotoxicity was specifically exhibited in HSPCs [30,37]. Overall, a classical animal model to induce significant hematotoxicity by benzene injection was successfully established in this study.

Previous research showed that miRNAs may participate in benzene-induced hematotoxicity in humans [29]. A research on patients with chronic benzene poisoning and healthy control showed that there was an aberrant expression of miRNAs in peripheral blood mononuclear cells. As such, we investigated differentially expressed miRNAs in HSPCs to reveal the molecular mechanisms of benzene-induced hematotoxicity. In this preliminary study, we identified a set of differentially expressed miRNAs between the benzene exposure and control groups. Subsequently, we analyzed eight of these miRNAs in Lin^−^ cells through qRT-PCR. The results of sequencing and qRT-PCR were in the same trend. We also investigated the expression of these eight miRNAs in the Lin^−^c-Kit^+^ cells after exposure to 150 mg/kg benzene through subcutaneous injection for 4 weeks. Of these miRNAs, five miRNAs, including mmu-miR-34a-5p, mmu-miR-342-3p, mmu-miR-100-5p, mmu-miR-181a-5p and mmu-miR-196b-5p, significantly changed in mice exposed to benzene. Evidently, quantification of miRNA levels through qRT-PCR revealed several differences between Lin^−^ cells and Lin^−^c-Kit^+^ cells in response to benzene.

Most of the miRNAs identified in this study have been shown to be dysregulated in various hematological malignancies [38,39,40,41,42,43,44,45,46]. For example, in a murine chronic lymphocytic leukemia (B-CLL) model, miR-34a expression was low in the preleukemic phase but remarkably increased in the leukemic phase. Similarly, the expression of miR-34a in patients with B-CLL was 4.6-fold higher than that of healthy controls [26]. MiR-129-5p and miR-100 were obviously different between patients with acute myeloid leukemia (AML) and normal controls [39,44]. However, the expression of miR-181a in different hematological malignancies were inconsistent. MiR-181a was upregulated in AML and myelodysplastic syndromes [47,48] but downregulated in multiple myeloma and chronic lymphocyte leukemia [49,50]. Furthermore, whether these miRNAs act as tumor suppressors or oncogenes remains unclear [44,46]. Therefore, we only described the aberrant miRNA expression profiles after benzene exposure in the present study. Further studies should be performed to verify the potential applications of these miRNAs in the prognosis and therapy of cancers.

Most of the miRNAs identified in this study have important roles in the regulation of hematopoiesis, such as in differentiation and proliferation of HSPCs. MiR-100 could promote the cell proliferation of promyelocytic blasts and block granulocyte/monocyte differentiation by targeting RBSP3 in AML [44]. Chen *et al.* [51] reported that miR-181 was preferentially expressed in B cells of bone marrow in mice; moreover, a tissue culture differentiation assay in mice showed that the fraction of B-lineage cells increased after ectopic expression in HSPCs. Another study revealed that miR-181 could effectively suppress the expression of Lin28 expression, disrupt the Lin28-let-7 reciprocal regulatory loop, upregulate Let-7, and eventually promote the differentiation of megakaryocytic. Nevertheless, miR-181 had no function on hemin-induced erythrocyte differentiation [52]. A previous microarray analysis showed that expressions of 11 miRNAs, including miR-196b, showed higher levels in the Lin^−^c-Kit^+^Sca-1^+^ compartment compared with those in total bone marrow cells; these miRNAs had functions to regulate stem cell homeostasis [53]. Meanwhile, the cluster of miR-144/451 was coexpressed from a common precursor transcript and functional cooperativity in mammalian erythropoiesis. However, an *in vitro* study showed that only miR-451 had functions in zebrafish erythropoiesis [23]. Another *in vivo* study confirmed that miR-451 played a positive role in the regulation of erythroid maturation in erythroid-differentiating K562 cells [54]. In this animal model, although the number of red blood cells significantly decreased in the benzene exposure group, the degree of decline was significantly less than that of white blood cells. No significant difference in miR-144/451 expression, which regulates the erythroid differentiation in Lin^−^c-Kit^+^ cells, was observed.

In the present study, miR-34a expression significantly changed at different levels in hematopoietic cells, including Lin^−^ cells and Lin^−^c-Kit^+^ cells after benzene exposure. The expression patterns are consistent with the results detected in peripheral blood cells of chronic benzene-exposed patients [29]. This finding confirmed that miR-34a may be involved in the regulation of chronic benzene poisoning.

This study provides valuable evidence on the function of miRNAs in hematopoiesis during benzene exposure. Further experiments should be performed to determine the nature of these miRNAs in the regulation mechanism of benzene toxicity.

## 4. Experimental Section

### 4.1. Reagents

Benzene, with a purity of ≥99.9%, was purchased from Sigma–Aldrich (St. Louis, MO, USA). A mouse HSPC Isolation Kit (Becton Dickinson, San Jose, CA, USA) was used for flow cytometric analysis and cell sorting.

### 4.2. Benzene Exposure

Male C57BL/6 mice were housed as previously described [55]. Normal diets and water were given *ad libitum* throughout the study period. Mice aged 6–8 weeks old were randomly divided into two groups. One group was exposed to benzene (150 mg/kg) through subcutaneous injection once every day, 5 days per week for 4 weeks. The other group was exposed to oil solution through subcutaneous injection and designated as the normal control group. At the beginning of exposure, there was no statistical difference in the body weight of mice between the two groups. All animal procedures were carried out according to the approved protocols of the local authorizing agency for animal experiments. The protocol of experiments was reviewed and approved by the Research Ethics Committee of the Southeast University (approval number: 20140168).

### 4.3. Organ Coefficient and Hematological Parameters

The body weights of mice were recorded twice a week during the whole exposure period and each mouse was weighed after exposure. At the end of the four weeks of exposure, liver, spleen, and thymus were harvested and weighed. We calculated the ratio between organ weight and body weight as organ coefficient. To detect hematological parameters, we collected 200 μL of peripheral blood from the orbital sinus of each mouse in a tube containing ethylene diamine tetraacetic acid (EDTA) after exposure. The sample was immediately analyzed using a Sysmex XE-2100 fully automatic hematology analyzer (Sysmex, Kobe, Japan). Total red blood cells (RBC), white blood cells (WBC), neutrophils (Neut), lymphocytes (Lym), platelets (Plt), the total hemoglobin (Hb), and the mean corpuscular volume (MCV) were measured.

### 4.4. Flow Cytometric Analysis

**Figure 5:**
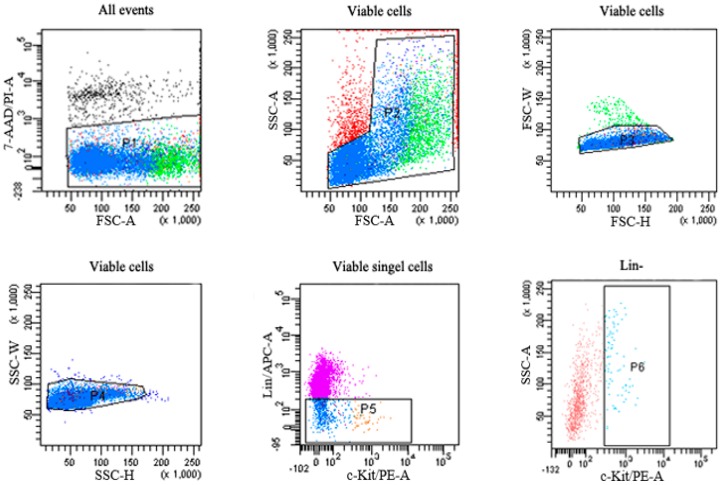
Gating strategy for detection and sorting various hematopoietic progenitor cells in the bone marrow by FACS. P1 is gated on the viable bone marrow cells from all events; P2 is gated on the P1-gated viable cells; P3 is gated on the P2-gated cells to remove doublets ; P4 is gated on the P3-gated cells to remove doublets; P5 is gated on Lin^−^c cells from P4-gated cells; P6 is gated on Lin^−^c-Kit^+^ cells from P5-gated cells.

Bone marrow cells were harvested from limb bones (including the humerus, tibiae, and femur) of each mouse as previously described [32,56]. Briefly, both ends of the bones were cut and bone marrow cells were flushed with buffer. After centrifugation at 250× *g* for 10 min, bone marrow cells were suspended and adjusted to 10^8^ cells/mL. To obtain a single-cell suspension, we filtered the cells through a strainer. For detection and sorting, the cells were blocked with Fc Block (Becton Dickinson, San Jose, CA, USA) for 10 min at 4 °C avoiding light. Then the cells were stained with specific antibodies (PE anti-mouse c-Kit, PE-Cy7 anti-mouse Sca-1, and APC mouse lineage antibody cocktail) or the corresponding isotype controls in the dark for 45 min at 4 °C. After washing with buffer twice and adding 7-amnioactinomycin D (7-AAD), the count for Lin^−^c cells and Lin^−^c-Kit^+^ cells in bone marrow were analyzed with an FACS Aria^TM^ II flow cytometer (Becton Dickinson). Primitive subpopulations were gated as shown in Figure 5. A total of 100,000 events were analyzed for each sample.

### 4.5. Sorting of HSPCs through Flow Cytometry and Total RNA Extraction

Lin^−^ and Lin^−^c-Kit^+^ cells were obtained with the FACS Aria™ II flow cytometer (Becton Dickinson, USA) to analyze different miRNA expression profiles between the control and benzene exposure groups. To obtain sufficient cells per group for Illumina sequencing and qRT-PCR analysis, we pooled Lin^−^ cells from two mice per group and Lin^−^c-Kit^+^ cells from three mice per group. After sorting, Lin^−^ cells as well as Lin^−^c-Kit^+^ cells were stored at −80 °C. Within 1 month, total RNA was lysed with Trizol (Invitrogen, USA). Subsequently, RNA was separated with chloroform, precipitated with isopropyl alcohol, washed with 75% ethanol for twice, and then dissolved in RNase-free water.

### 4.6. Illumina Sequencing of miRNAs

The integrity of RNA was detected using the Agilent 2200 TapeStation (Agilent Technologies, Santa Clara, CA, USA). Each sample presented an RNA integrity number equivalent to high than 7.0. The purity of RNA was determined with the Nanodrop ND-1000. For all RNA samples, the ratios of A260:A280 and A260:A230 should higher than 1.8 and 2.0, respectively. Total RNA was sent to Guangzhou RioBio Co., Ltd. (Guangzhou, China) to sequence miRNA expression profiles with a Hiseq 2500 Analyzer (Illumina, San Diego, CA, USA). Briefly, RNAs were ligated with 3′ RNA adapter, followed by 5′ adapter. Adapter-ligation RNAs were subjected to RT-PCR and amplified at a low cycle. PCR products were size-selected through PAGE according to the instructions in the TruSeq^®^ small RNA sample prep kit (Illumina, San Diego, CA, USA). The Agilent 2200 TapeStation was used to evaluate the purified library products. The products were then diluted to 10 pM for cluster generation *in situ* on the HiSeq 2500 single-end flow cell and then sequenced (1 × 50 bp). To obtain miRNA profiles that were significantly induced or suppressed by benzene, we set the fold change threshold of benzene exposure at |log_2_ (Fold Change)| >1 as compared to control for further analysis. Aberrant expression of miRNAs was determined using ANOVA.

### 4.7. Verification of Sequencing through qRT-PCR Analysis

We selected eight miRNAs that significantly changed based on the sequencing result and have been reported to be involved in processes related to hematopoiesis, and hematological malignancies including leukemia [23,24,42,44,52,53,57]. For qRT-PCR assay, total RNA from the pooled Lin^−^ cells and Lin^−^c-kit^+^ cells were obtained as mentioned in sorting of HSPCs through flow cytometry. We used a SYBR^®^ PrimeScript^TM^ miRNA RT-PCR kit (TaKaRa, Dalian, China) to detect and quantify these eight miRNAs. Briefly, the total RNA was transcribed into cDNA which was used as template for miRNA PCR array. Reverse-transcription reaction was conducted in 10 μL solution containing 5 μL of 2× miRNA reaction buffer mix, 2 μL of RNase-free water, 1 μL of miRNA PrimeScript RT enzyme mix, 1 μL of 0.1% BSA, and 1 μL of total RNA extracted from Lin^−^ cells. The mixture was kept at 37 °C for 60 min and 85 °C for 5 s. The real-time PCR reaction was performed in a final volume of 10 μL comprising 5 μL of 2× SYBR premix Ex Taq II, 3 μL of RNase-free water, 1 μL of cDNA, 0.4 μL of miRNA qPCR primer, and 0.2 μL of ROX. The real-time PCR protocols included 95 °C for 30 s; 40 cycles of 95 °C for 5 s and 60 °C for 30 s; and a 4 °C holding period. All the samples were analyzed in triplicate. To calculate the relative gene expression with 2^−ΔΔCt^ method, miRNA (U6) was used as the reference gene. To ensure specificity, the PCR products were analyzed with melting curve. The expression data for miRNA were acquired and analyzed with the StepOnePlus^TM^ real-time PCR systems and StepOne Software v2.2.2. (Applied Biosystems, Foster, CA, USA).

### 4.8. Statistical Analysis

Data are showed as mean ± SD. Results were first evaluated for homogeneity of variance. If the variables were homogeneous, a one-way ANOVA was performed followed by an independent sample Student’s *t* test. If the variances were not equal, the non-parametric Mann-Whitney test was employed. We considered *p* < 0.05 to be significant. All results were analyzed by SPSS version 13.0 (SPSS, Chicago, IL, USA).

## 5. Conclusions

This study revealed that benzene exposure causes severe hematotoxicity and alters miRNA levels in HSPCs. The miRNA expression pattern suggests that benzene-exposed mice may undergo a series of epigenetic changes involved in regulating critical steps in the formation of HSPCs.
